# A Rare Presentation of Cardiac Aspergilloma in an Immunocompetent Host: Case Report and Literature Review

**DOI:** 10.7759/cureus.4784

**Published:** 2019-05-30

**Authors:** Abdullah M Pervaiz, Salman A Bangash, Raheel Akhtar, Zain Wahab, Hyder Bangash

**Affiliations:** 1 Internal Medicine, University of Massachusetts Medical School - Baystate Medical Center, Springfield, USA; 2 Internal Medicine, Doctors Hospital / University of Texas Rio Grande Valley, Edinburg, USA; 3 Internal Medicine, Windsor University School of Medicine, Basseterre, KNA; 4 Internal Medicine, Kingsbrook Jewish Medical Center, New York, USA; 5 Psychology, The University of Texas at Austin, Austin, USA

**Keywords:** aspergilloma, cardiac aspergilloma, infective endocarditis, aspergillus, immunocompetent, cerebral emboli

## Abstract

Cardiac aspergilloma is exceptionally rare with only a handful of cases reported and majority of them being in immunocompromised patients. Here, we present a case of cardiac aspergilloma involving the right and left ventricle in an immunocompetent patient that initially presented with acute limb ischemia. He was later found to have a cardiac mass with histopathological diagnosis confirming Aspergillus species. Despite aggressive medical and surgical interventions, the patient had an unfavorable outcome due to low suspicion of invasive fungal endocarditis given his immunocompetent status. Cardiac aspergilloma should remain in the differential diagnosis of immunocompetent patients as early clinical suspicion will result in early treatment and decreased mortality. Novel therapies are required to decrease mortality in the future from this fatal disease.

## Introduction

Aspergillus is a common fungus that is present in the environment and is known to cause fatal disease, particularly in immunocompromised hosts. Fungal endocarditis is rare and accounts for <2% documented cases of infective endocarditis of which only 20-25% are caused by aspergillus species [1]. Invasive aspergillus involving heart valves and cardiac chambers is exceptionally rare with only a handful of cases reported and majority of them being in immunocompromised patients. Here, we present a case of cardiac aspergilloma involving the right and left ventricle in an immunocompetent patient that initially presented with acute limb ischemia.

## Case presentation

A 19-year-old male with no known past medical history presented to the hospital with a two-day history of right-sided leg pain with yellowish to bluish discoloration. The patient was tachycardic, normotensive, and afebrile. On examination, he was found to have a cool right lower extremity, delayed capillary refill, and diminished femoral, popliteal, posterior tibial, and dorsalis pedis pulses. The patient had a normal appearance of the contralateral extremity with palpable pulses. On auscultation of his heart, he did not have any murmurs, rubs, or gallops. No focal neurological deficits were appreciated on examination. Laboratory workup revealed a normal complete blood picture and comprehensive metabolic panel, erythrocyte sedimentation rate 115, c-reactive protein 13.5, procalcitonin 0.518, and negative serology for HIV, hepatitis B, and C. Autoimmune workup with antinuclear antibody (ANA), anti-double stranded DNA (dsDNA), complement C3/C4, and antiphospholipid antibodies was also negative. Electrocardiogram showed sinus tachycardia without acute ischemic changes. Chest X-ray did not show an acute cardiopulmonary process. Bilateral arterial and venous dopplers were done which showed a thrombus in the right femoral artery.

**Figure 1:**
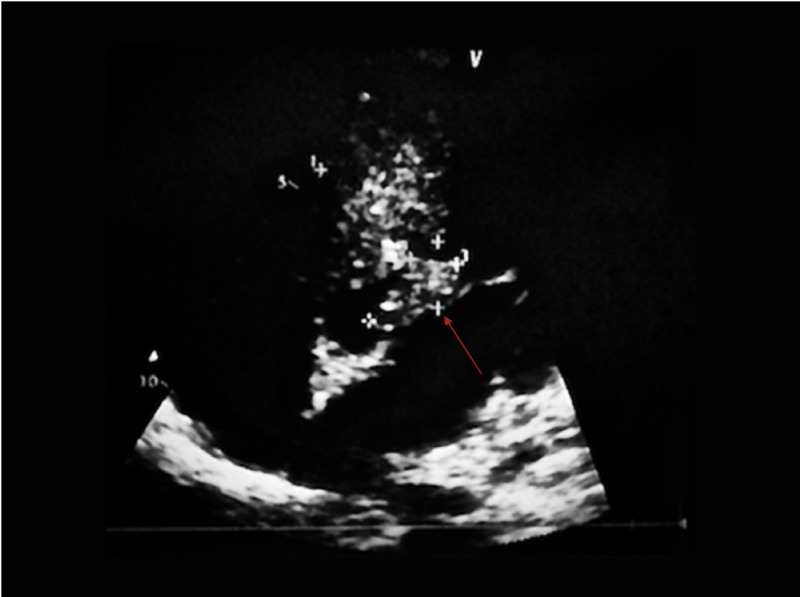
Parasternal long axis view on transthoracic echocardiogram showing a mass extending into the left ventricular outflow tract (LVOT).

The patient was taken to the operating room and successfully underwent right femoral embolectomy for acute limb ischemia. On the first postoperative day, the patient developed blurry vision in his left eye. A CT head without contrast showed no evidence of acute intracranial pathology. Due to concerns for cardiac source of systemic embolization a transthoracic echocardiogram (TTE) was done which showed a 12 × 10 mm mass hanging from the sub-aortic septum extending into the left ventricular outflow tract (LVOT) (Figure 1).

There was clinical suspicion for a cardiac rhabdomyosarcoma so further imaging was obtained to delineate the mass. Cardiac MRI showed a 37 x 45 mm complex irregular mass in the basal septum, growing towards both ventricles, eroding the left ventricle (LV) and further bifurcating into two linear extensions. One of the linear structures was freely mobile in the LVOT. The area of the mass in the right ventricle (RV) was calculated at 15.5 cm^2^ with fronds protruding into the RV cavity. MRI findings were read as consistent with sarcoma and recommended further work with a biopsy for confirmation. The patient subsequently underwent excision of the mass and the specimen was sent for histopathology.

**Figure 2:**
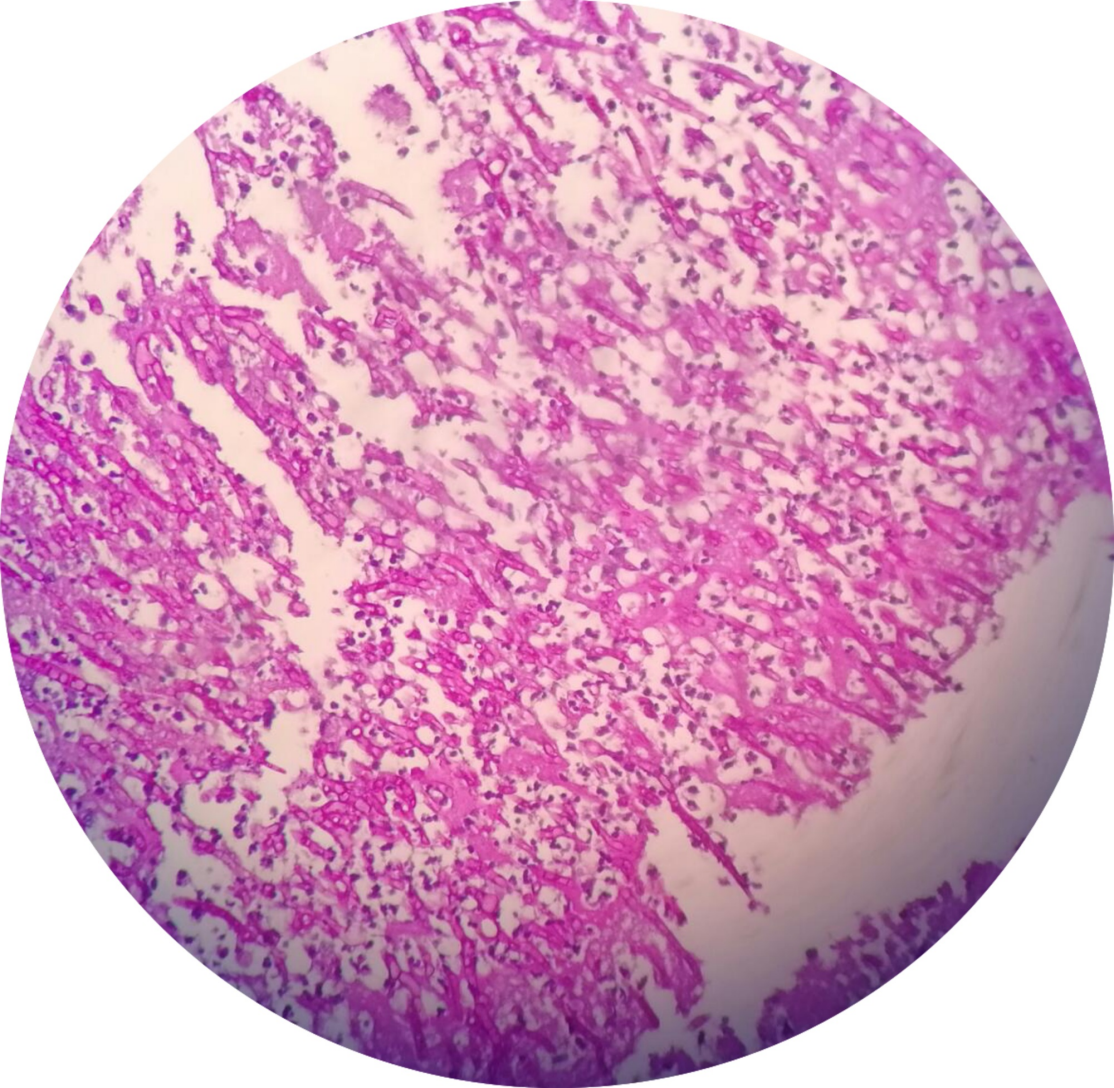
Histological examination showing numerous septate and branching fungal hyphae consistent with Aspergillus species.

The patient was extubated to nasal cannula within 12 hours postoperatively. Histopathology of the tissue obtained in the operating room showed numerous septate and branching fungal hyphae which were consistent with Aspergillus species (Figure 2). Infectious disease was consulted and he was initiated on liposomal amphotericin B. On his first post-operative day, he developed an acute change in his mental status and was subsequently found to have septic embolus to his brain on MRI. The patient was made comfort measures only and passed away shortly after.

## Discussion

Aspergillus is a common fungus that is present in the environment and easily inhaled; however, healthy individuals have a natural immunity to the aspergillus spores. Aspergillus infections account for 0.1-3.9% of annual cases in immunocompromised patients, out of which only 0.7-6% of patients develop cardiac involvement due to the fungal infection [2]. The common predisposing risk factors of an Aspergillus infection are immunocompromised status, damaged lungs, prosthetic valves, rheumatic heart disease, long-term placement of a central venous catheter, total parenteral nutrition, and IV drug use [3-4]. Cardiac aspergilloma as presented in our case is a rare condition that is not associated with any of the above predisposing risk factors, this unique presentation is only seen in 3.4% of total cases and has not been commonly reported in literature [5].

The common etiologic agents of infective endocarditis (IE) are bacteria and fungi. Candida followed by Aspergillus are the two most common fungi responsible for fungal endocarditis [3]. Cardiac fungal infections with Aspergillus species have a high mortality rate due to the nature of the disease, difficulty in early diagnosis, and high rates of systemic embolization. In patients with aspergillus endocarditis, approximately 50% of blood cultures yield negative results as Aspergillus species is not easily detected [3]. TTE is the first diagnostic test for patients suspected of having IE with a sensitivity of 75% and specificity approaching 100%. However, a negative test result does not preclude the diagnosis and a transesophageal echocardiogram (TEE) is warranted [6]. Fungal vegetations are typically characterized as large, bulky, and highly mobile, with an increased risk of embolic events most commonly affecting the brain, as seen in our case. Cardiac MRI is a noninvasive method superior to TTE in diagnosis, differentiation, and detection of extension of cardiac masses [7]. However, the test is not always required in the diagnosis of cardiac aspergilloma. In our patient, TTE showed a large, mobile cardiac mass for which the differential diagnosis included fungal etiology, thrombus, primary cardiac tumor, myxoma, metastatic lesion, and lymphoma. In our patient, there was initial suspicion of cardiac rhabdomyosarcoma after which a cardiac MRI was obtained to further delineate the mass. Final diagnosis is best achieved by excision and biopsy of the mass. In our patient, the histologic examination revealed fungal septate hyphae confirming Aspergillus species.

A multi-disciplinary effort involving cardiology, infectious disease, and cardiac surgery is required in managing patients with cardiac aspergilloma given the complexity and high mortality associated with the disease process. Most recent reviews as well as Infectious Diseases Society of America (IDSA) guidelines favor both pharmacological and surgical interventions for treatment of Aspergillus endocarditis early in the disease course to help decrease poor outcomes and mortality which include compromising cardiac function and the risk of embolic events [8]. Per IDSA, the recommended initial antifungal agents are liposomal amphotericin B or voriconazole, followed by lifelong antifungal treatment postsurgical intervention [9]. Yuan has proposed using caspofungin in addition to voriconazole and amphotericin, however, the use of combining antifungal therapies has not been shown to be superior to monotherapy [3,9]. In the described case, the patient underwent surgical excision of the mass and was treated with systemic liposomal amphotericin B late in his hospital course, as there was a low clinical suspicion of invasive aspergillus given his immunocompetent state. On review of the current literature, only a small number of cases of cardiac aspergilloma have previously been reported (Table 1) [4, 10-16].

**Table 1:** Review of current literature describing reported cases of cardiac aspergilloma.

Author (Year)	Patient characteristics	Findings
Chen-Milhone et al. (2018) [4]	62-year-old immunocompromised male with a past medical history of prostatic adenocarcinoma on chemotherapy.	Cardiac aspergilloma involving the native tricuspid valve, right atrium and ventricle.
Soman et al. (2014) [10]	22-year-old immunocompetent male with a past medical history of hepatitis B.	Aspergillosis extensively involving all four chambers, the tricuspid and mitral valves of the heart.
Paul et al. (2012) [11]	60-year-old female with leukemia who had undergone allogeneic stem cell transplantation	Invasive aspergillosis of the interventricular septum.
Alvarez et al. (2004) [12]	12-year-old female with acute lymphoblastic leukemia on chemotherapy.	Left ventricular pedunculated mass that was detected by echocardiographic study; at surgery, the presence of Aspergillus terreus was confirmed.
Chou et al. (2013) [13]	27-year-old man receiving chemotherapy for acute myeloid leukemia.	A large right ventricular mass was detected on transthoracic echocardiogram after surgical excision a specimen culture yielded Aspergillus flavus.
Singla et al. (2011) [14]	26-year-old male with a past medical history of polysubstance abuse who was being treated for complications of acute pancreatitis.	A large mobile tricuspid valve aspergilloma obstructing the right ventricular inlet, diagnosed incidentally on the second postoperative day after laparoscopic pancreatic abscess drainage.
Cishek et al. (1996) [15]	40-year-old woman with a 12-year history of severe ulcerative colitis unresponsive to treatment on high doses of prednisone and 6-mercaptopurine.	Aspergillosis invading the myocardium and causing widespread necrosis and infarction.
Dang-Tran et al. (2014) [16]	68-year-old male liver transplant patient on immunosuppressive therapy.	Isolated Aspergillosis Myocardial Abscesses

Majority of the reported cases of cardiac aspergilloma have occurred in immunocompromised patients and rarely in healthy individuals as observed in our patient. In all of the cases except for one, the outcome was unfavorable resulting in the death of the patient days or weeks after diagnosis. Fungal endocarditis is associated with a mortality rate of at least 50% with an average survival period of 11 days with a 30% recurrence rate in survivors [17]. Unfortunately, our patient developed a septic embolus to his brain which ultimately led to his demise.

## Conclusions

Cardiac aspergilloma is a rare disease with a high mortality rate despite aggressive pharmacological and surgical interventions. It is rarely reported in immunocompetent patients and carries a high mortality rate and often leads to unfavorable outcomes. Cardiac aspergilloma should remain in the differential diagnosis of cardiac masses in immunocompetent patients as early clinical suspicion will result in early treatment and decreased mortality. Survivors should be closely followed due to high recurrence rates.
